# The general practice workforce crisis in Europe: scale of the challenge and available policy responses

**DOI:** 10.1016/j.lanprc.2026.100166

**Published:** 2026-06

**Authors:** Giuliano Russo, Martin McKee, Tomas Zapata, Alba Llop Girones, Sulakshana Nandi, Sara Ares-Blanco

**Affiliations:** a Wolfson Institute of Population Health, Queen Mary University of London, London, UK; b Department of Health Services Research and Policy, London School of Hygiene & Tropical Medicine, London, UK; c WHO Regional Office for Europe, Copenhagen, Denmark; d Federica Montseny Health Centre, IiSGM, SERMAS, Madrid, Spain

## Abstract

General practice across the European region faces a systemic workforce crisis that threatens patient access to health services as well as the coordination, continuity, and equity of care within European health systems. In this Health Policy paper, we use an analysis of the WHO/Europe-Eurostat-Organisation for Economic Co-operation and Development (OECD) Joint Questionnaire on Non-Monetary Health Care Statistics dataset for 43 countries, evidence from a dedicated research collection, and a rapid review of policy interventions to assess the scale, drivers, and solutions to general practitioner (GP) shortages. Although headcounts have increased since 2010, GP density has stagnated, and effective full-time equivalent capacity has declined due to ageing, early retirement, part-time work, migration, administrative burden, and private-sector competition. We argue that incremental responses are insufficient and propose a coordinated, lifecycle-based strategy spanning training, recruitment, retention, and late-career roles. Key actions include expanded training pathways, improved employment conditions, rural and return-migration incentives, expanded multidisciplinary teams, reduced administrative workload, and full-time equivalent-sensitive workforce monitoring linked to modernised funding and contracting. Tailored national implementation within a shared European framework will be essential to rebuild sustainable GP capacity.

## Introduction

Across Europe, general practice and family medicine are facing a profound and escalating workforce crisis. Although longstanding challenges in recruiting and retaining general practitioners (GPs) have been documented for decades,^1^ the situation has intensified in the aftermath of the COVID-19 pandemic. Health systems in almost every European country now report worsening shortages of primary care physicians, longer waiting times, and growing political alarm over the financial sustainability of comprehensive primary care. WHO analyses warn that the European region is experiencing a multilayered, slow-burning primary care crisis, marked by simultaneous pressures on demand, supply, and health system governance.^2^

Media reports have amplified these concerns. *Politico* has asked why Europe is running out of doctors, particularly for countries like France, Germany, and the UK.^3^ In the UK, evidence given to a parliamentary committee described general practice as being at breaking point, with patients struggling to obtain timely appointments and GPs reporting untenable workloads and burnout.^4^ Professional organisations echo these concerns: the European Association of Senior Hospital Physicians, Conseil Européen des Ordres des Médecins, Standing Committee of European Doctors, European Junior Doctors, European Medical Students’ Association, European Federation of Salaried Doctors, European Union of General Practitioners/Family Physicians, and the European Union of Medical Specialists report that up to 43% of doctors experience symptoms of burnout, with excessive workloads and unsafe staffing among the most commonly cited drivers.^5^ A WHO Europe study on health workers’ mental health in the EU found that one in three doctors and nurses report symptoms of depression or anxiety.^6^

This sense of urgency is validated by emerging research. A comparative analysis of European countries^2^^,^^7^, ^8^, ^9^, ^10^, ^11^, ^12^ shows that no system, whether organised as a tax-funded National Health Service (NHS), a social health insurance model, or a post-communist hybrid, fully aligns with WHO benchmarks for strong primary care, and nearly all face substantial GP shortages and maldistribution.^13^ The WHO Europe report on health workforce migration shows that doctors from eastern and southern European countries migrate to wealthier EU member states in western and northern Europe, further deepening regional inequalities in access to primary care.^14^

The UK offers a powerful illustration of the structural weaknesses emerging across Europe. Workforce data show that although GP training numbers are increasing, the rate at which newly trained doctors join the NHS as fully qualified GPs is declining.^12^ Fewer than two-thirds (62%) of GP trainees take up a qualified GP role within 2 years of completing training, and the figure has fallen further for the most recent cohorts. Similar issues are visible across Europe: in Germany, Switzerland, Spain, and Bulgaria, the majority of primary care physicians are approaching retirement age; Greece has a high overall doctor-to-population ratio but a persistently low proportion of GPs within that workforce; and in Kazakhstan, Serbia, and Romania, shortages are compounded by uneven geographical distribution and weak governance structures.

Politicians across Europe increasingly acknowledge the severity of the crisis. At a 2025 European Parliament hearing, members of the European Parliament described the workforce crisis as a shared European problem, emphasising that even wealthier countries are struggling to recruit and retain enough doctors and are calling for coordinated EU-level action to stabilise the health workforce.^15^ The urgency of reform is reflected in calls for greater adherence to the Working Time Directive, improved postgraduate training, better mental health support for GPs, and investment in community-based models that can reduce avoidable demand for hospital care.

Taken together, these trends point to a continental crisis with deep structural roots. This paper examines the scale, nature, and drivers of the GP workforce crisis in the European region (as defined by WHO), drawing on comparative evidence, national case studies, and cross-sector perspectives to inform policy solutions commensurate with the magnitude of the challenge.

## Methods

While acknowledging variation in the role of GPs across health systems, this Health Policy paper defines GPs as medical specialists trained to diagnose and manage undifferentiated health problems across all age groups; provide acute, chronic, and preventive care; integrate biomedical, psychological, and social factors in clinical decision-making; and coordinate care across services (panel). Although the WHO European region includes 53 member states, the analysis focuses on 43 countries that regularly report data to the WHO/Europe-Eurostat- Organisation for Economic Co-operation and Development (OECD) Joint Questionnaire on Non-Monetary Health Care Statistics health workforce dataset.

Panel **General practice in Europe: evolution, variation, and the need for a unified operational definition**

The role of a general practitioner (GP) in Europe has transformed significantly since the mid-twentieth century. As medical science expanded after the 1950s and specialisation intensified, it became clear that no doctor could master all areas of medicine. By the 1970s, experts recognised that GPs needed to work more systematically, use increasingly sophisticated diagnostic and therapeutic tools, and maintain strong patient relationships within an increasingly technical environment. They also had to coordinate care with a widening range of non-medical professionals essential to patient support.

By the early 1990s, general practice had become a clearly defined pillar of primary care, although organised very differently across Europe. The largest study to date,^16^ undertaken in 1993, of 7233 GPs across 30 European countries, revealed major differences in first-contact care, medical procedures, disease management, and prevention. GPs had broader roles in countries with gatekeeping systems or self-employment, whereas those in eastern Europe and some Mediterranean countries had more restricted-service profiles. Overall, general practice was more comprehensive where structural features supported stronger GP involvement.

From the 2000s onwards, the profession became more formalised. Successive definitions, issued by the World Organization of Family Doctors (WONCA) Europe in 2002, 2005, 2011, and 2023,^17^ established general practice as a distinct speciality, emphasising person-centredness, continuity, comprehensiveness, and community orientation.

In practice, however, the work of GPs in Europe varies so widely—between countries, within countries, and even between neighbouring practices—that creating a single definition that genuinely encompasses this diversity is inherently difficult. Some systems separate adult and childcare; others rely on broad family-practice models. Levels of digitalisation, multidisciplinary team working, and clinical scope differ enormously. Although the WONCA Europe document offers an ambitious and detailed definition, it is constrained by these variations and reflects an idealised standard that many systems cannot meet. Its breadth becomes a limitation; by trying to include everything, it struggles to describe what GPs consistently are across Europe. We thus propose a high-level definition that does not replicate WONCA’s in detail but is deliberately broad enough to cover the full spectrum of practice styles, organisational models, and system contexts. In light of these considerations, we propose the following definition:

GPs are medical specialists with the skills and expertise across the entire spectrum of medicine needed to diagnose and manage undifferentiated problems across all ages; make decisions with limited information; address acute, chronic, and preventive needs, integrating physical, psychological, social, and environmental factors in consultations; and communicate effectively with all those involved in the care of the patient.

In the datasets we used to graph the evolution of the medical workforce in Europe, definitions of GPs are based on the Joint Questionnaire on Non-Monetary Health Care Statistics developed by the WHO Regional Office for Europe, Eurostat, and the OECD,^18^ which provides harmonised concepts to support cross-country comparability of health workforce data. In such databases, the primary care doctor’s category includes GPs, district medical doctors or therapists, family medical practitioners, and medical interns or residents specialising in general practice. Paediatricians and other generalist (non-specialist) medical practitioners are excluded. Although in some countries, general practice or family medicine is formally recognised as a medical specialty, these practitioners are always classified as GPs for statistical purposes. In line with the System of Health Accounts 2011, offices of general medical practitioners (HP.3.1.1) comprise establishments of doctors holding a medical degree who are primarily engaged in the independent practice of general medicine.

This paper draws on three complementary sources of evidence: (1) secondary analysis of an international medical workforce dataset; (2) empirical findings from a coordinated collection of studies on the GP workforce crisis in Europe;^2^ and (3) a rapid review of policy interventions aimed at strengthening the primary care medical workforce.

### Analysis of workforce datasets

Regional workforce trends were analysed using data from the WHO/Europe-Eurostat-OECD Joint Questionnaire on Non-Monetary Health Care Statistics,^18^ an annual standardised dataset compiling national health workforce statistics across the WHO European region. Data were obtained in February, 2026, from the Unit for Health Workforce and Service Delivery at the WHO Regional Office for Europe in Copenhagen (Denmark).

The analysis focused on two physician categories defined in the Joint Questionnaire framework: GPs and other generalist (non-specialist) medical practitioners. The GP category includes physicians who provide comprehensive primary medical care to individuals and families, such as family doctors, district medical doctors, and GP trainees. Paediatricians and other specialists not practising as family doctors are excluded. The other generalist category includes physicians practising without a defined speciality but not classified as GPs, such as generalists working in hospital settings or doctors without a designated speciality area.

**Figure 1:**
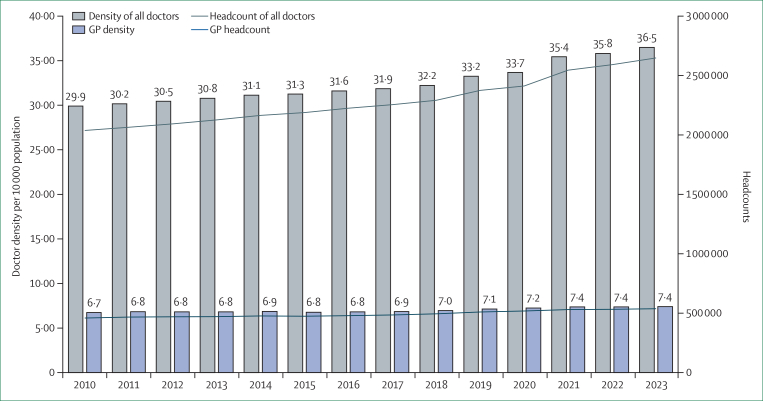
Evolution of doctor headcount and density between 2010 and 2023 (per 10 000 population, for all doctors and for GPs) Data are from the WHO/Europe-Eurostat-OECD Joint Questionnaire on Non-Monetary Health Care Statistics, 2026. Total medical doctors include generalist medical practitioners and specialist medical practitioners. Chart includes 43 countries in the WHO European region with sufficient data, and excludes Albania, Georgia, Kyrgyzstan, Monaco, North Macedonia, Russia, San Marino, Slovakia, Tajikistan, and Ukraine. Missing headcounts are filled with interpolation or extrapolation as necessary. GP=general practitioner. OECD=Organisation for Economic Co-operation and Development.

For doctor headcount and density (figure 1), the data cutoffs applied were January, 2010, to December, 2023, that is, all the years for which complete datapoints were available in the dataset. For GPs as proportion of total medical workforce, the cutoff was 2014–23—all the available years with complete datapoints. All variables were analysed as headcounts of practising physicians reported through national administrative workforce statistics submitted to the database, ensuring cross-country comparability through standardised definitions and reporting procedures. Population denominators used to calculate workforce density indicators were derived from UN World Population Prospects mid-year population estimates. In the Joint Questionnaire, regional densities were calculated via population-weighted aggregation to ensure that the density reflects the true availability of health workers for the total population within a region rather than merely providing an unweighted average of national policies; this approach was followed to ensure that small countries with high health worker density would not skew the average upwards, masking shortages in neighbouring large countries.^18^

### Review of published literature

First, the paper draws on empirical evidence from 13 peer-reviewed studies published in a coordinated academic collection examining the GP workforce crisis across European health systems.^2^^,^^7^, ^8^, ^9^, ^10^, ^11^, ^12^^,^^19^, ^20^, ^21^, ^22^ Such studies were included in our methods as a purposively selected, peer-reviewed thematic series generated through a targeted call for papers led by an expert editorial group and focused on the WHO European region. The papers from the collection underwent standard peer review and were curated to ensure thematic and methodological coherence, with a strong emphasis on post-COVID-19 developments, cross-country comparison, and policy relevance. The inclusion of this collection, therefore, provided a set of contemporaneous and methodologically aligned studies that enabled more robust comparability across countries within a common analytical framework; this collection complemented the broader rapid review by contributing greater depth, conceptual coherence, and policy granularity, thereby strengthening the overall synthesis without duplicating its breadth.

Second, a rapid review of the literature was conducted to identify policy approaches to strengthen the primary care physician workforce. The review followed methodological guidance from the Cochrane Rapid Reviews Methods Group^23^ and published reporting guidelines for rapid reviews.^24^ Searches were conducted from Feb 1, 2026 to March 25, 2026 in MEDLINE, Scopus, Web of Science, and EconLit, using combinations of keywords related to primary care physicians (eg, “GP” and “family physician”), workforce issues (eg, “recruitment”, “retention”, and “distribution”), and policy interventions (eg, “policy”, “programme”, and “reform”). Searches were restricted to peer-reviewed articles published in English between Jan 1, 2020 and Dec 31, 2025 to include the post-COVID literature and were conducted by the lead author (GR).

Studies were included if they examined policies or system reforms targeting the recruitment, distribution, retention, or working conditions of primary care physicians and reported empirical or systematic analyses. Commentaries and studies that did not directly address physician workforce policies were excluded. Titles and abstracts were screened for relevance, followed by a full-text review of eligible articles. Data were extracted using a structured template capturing study characteristics, policy type, geographical context, and workforce outcomes.

Findings were synthesised narratively to identify common policy approaches relevant to strengthening GP workforce capacity.

In the following sections, we reflect on findings from our descriptive analysis of the Joint Questionnaire dataset and review 61 papers reporting evidence on the GP crisis in Europe (appendix pp 1–5). These studies comprise quantitative (n=13) and qualitative research (n=12), reviews (n=10), surveys (n=9), conceptual analyses (n=6), policy analyses (n=5), and case studies and mixed-methods studies (n=3 each). Most papers focused either on Europe as a whole (n=19) or on a global context that included Europe (n=3). The remainder examined specific countries, including the UK (n=18), Ireland (n=3), and Belgium, Germany, Portugal, Spain, and Sweden (n=2 each).

## The contours of a crisis: evolution of the GP workforce in Europe

Although our datasets show that the overall numbers of GPs in Europe are at an all-time high, a closer analysis shows that, in the last decade (2014–24), both the ratio of GP per capita in the European region has not increased despite the growing demands, in fact, the ratio has decreased in 17 countries, and; GPs are becoming a shrinking proportion of the medical workforce.

Across 43 countries in the European region, the headcount of GPs increased from 459 152 in 2010 to 537 457 in 2023 (17%). However, the density of GPs in the region increased by 10% between 2010 and 2023 (from 6·7 to 7·4 per 10 000 population), less than half the increase in the overall density of doctors (22%).

There are large disparities in the density of GPs across the European region, with countries such as Portugal displaying 29·5 GPs per 10 000 population, whereas Poland has just 2·4 GPs per 10 000 population.

GPs account for 20% of the physician population in the European region, down from 23% in 2010. Again, cross-country differences are very large, with countries like Portugal, where GPs account for 49% of all physicians in 2023 (up from 46% in 2014), and Greece, where they represent just 7% of the physician workforce (up from 6% in 2014).

**Figure 2:**
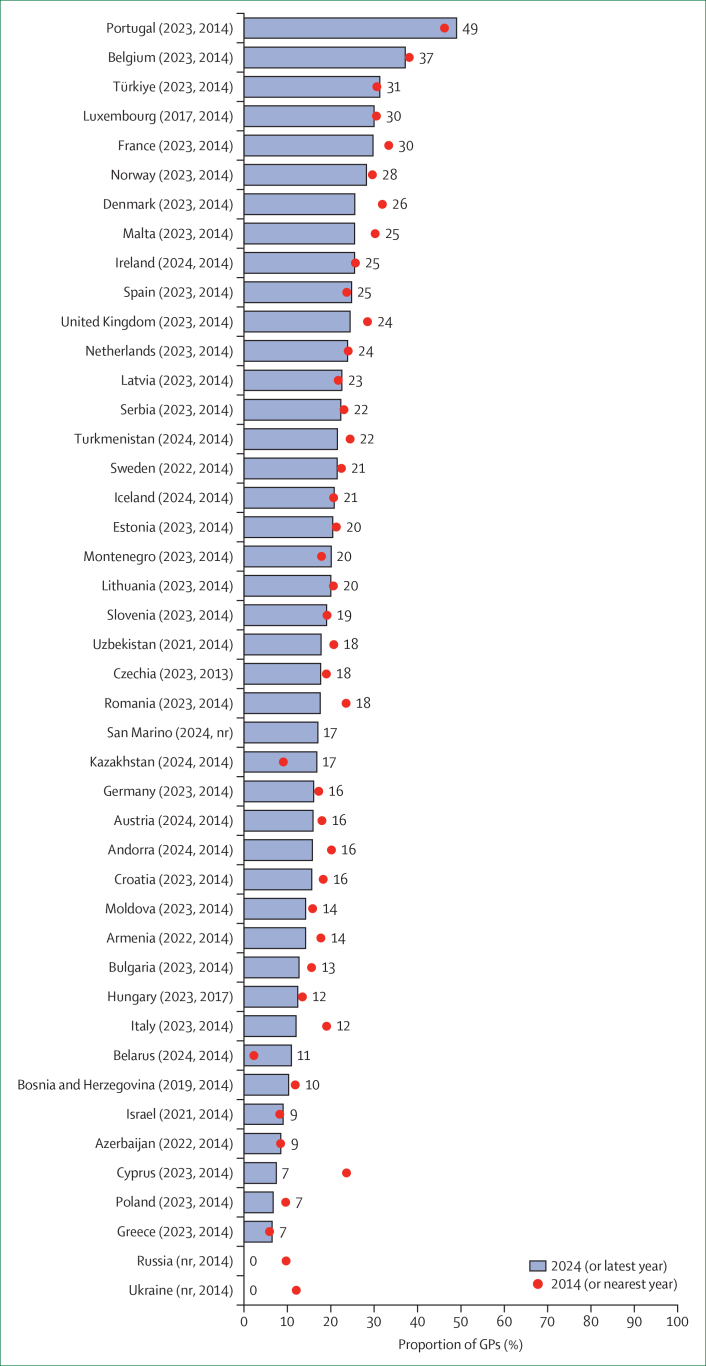
GPs as a proportion of the total physician workforce in 43 selected countries of the WHO European region in 2014 and 2024 (or closest available complete year) Data are from the WHO-Eurostat-OECD Joint Questionnaire, 2026. GP=general practitioner. NR=no record available. OECD=Organisation for Economic Co-operation and Development.

The proportion of GPs among total physicians went down markedly in the last decade—from 37% in 2014 to 30% in 2024, with countries like Italy recording the largest decreases (from 19% to 12%), followed by Romania (from 24% to 18%), France (from 33% to 30%), the UK (from 29% to 25%), and Denmark (from 32% to 26%; figure 2).

Over the past decade, Europe’s GP workforce has entered a period of simultaneous demand surge and supply fragility. Across diverse health system models, including the Beveridge model, social health insurance, and mixed post-communist systems, countries report deteriorating access to first-contact care, rising waiting times, and growing political pressure to fix general practice. Comparative assessments suggest that no country fully aligns with WHO’s attributes for strong primary care;^11^ shortages, maldistribution, and governance gaps are common, even in systems historically considered robust. The crisis is layered: ageing populations and multimorbidity increase demand; the pandemic has intensified the workload; and supply is constrained by retirement waves, emigration, and declining retention of newly qualified doctors.^2^

### Supply under strain: declining attractiveness, fewer joiners, earlier exits, and reduced intensity

Possibly reflecting scarce exposure during medical education, general practice is perceived by students as one of the least attractive medical specialities.^25^, ^26^, ^27^ This perception has been attributed to several factors, including the insufficient recognition of primary care as a speciality in some countries, comparatively lower remuneration, the dominance of treatment-centred and hospital-centred medical cultures, and few opportunities for career progression and research.^28^ Although medicine remains a popular option, with numerus clausus policies restricting the number of new entrants, the number of medical students choosing general practice has been declining.

Evidence from Czechia^20^ focuses on the other end of the pipeline: working-life expectancy for primary care physicians at age 50 is approximately 20 years (women 20; men 21) and fell by approximately 1 year between 2014 and 2022, with signs of earlier exits among some cohorts during the pandemic era and after administrative changes (eg, e-prescription obligations). These findings challenge the assumption that physicians will naturally extend careers to offset shortages.

Although workforce contractions are typically attributed to different combinations of declining entry and earlier exit, Portugal highlights a third dynamic: even where the absolute number of enrolled patients fell slightly and hiring continued, the coverage rate deteriorated after 2019 because GP full-time equivalents (FTEs) declined and the average panel list size per GP contracted (the phenomenon was called productivity effect by the authors of the study).^8^ Decomposition of 2009–23 trends shows that a 5% decline in GP FTEs and a progressive reduction in patients per GP together outweighed the demand-side relief from fewer enrollees, raising the number of residents without an assigned family doctor to 17% in 2023. In short, the system is losing capacity through fewer hours per clinician and structural shifts (eg, panel-size rules and case-mix complexity) that reduce the volume each GP can safely carry. An analysis of GP headcount and FTE in the UK corroborates these findings,^29^ showing that, between 2015 and 2024, NHS general practice lost one FTE GP annually for every five additional General Medical Council-licensed GPs. The gap between licensed GPs and those working in NHS general practice was largest among female, young (those aged 30–39 years), and UK-qualified GPs and those based in London and the Southeast of England. By 2024, each FTE GP served roughly twice as many NHS patients as an FTE consultant, indicating a widening imbalance between primary and secondary care workforce capacity.

### Cross-border pressures: mobility and misaligned incentives

Intra-European mobility compounds shortages, especially in countries with lower pay, precarious contracts, or restricted career prospects. A mixed-methods study of Spanish-trained GPs working abroad^7^ found salary, job insecurity, excessive workload, poor governance, and inflexibility as the main push factors; notably, nearly half would consider returning if conditions improved, an important policy lever for retention and repatriation strategies. The same pattern emerges in system-level reviews: governance and labour-market misalignments, such as the legacy of historical numerus clausus policies, hospital-centric training cultures, and undervalued primary care, hinder both recruitment and retention, particularly in rural or deprived areas.

### Workload, work design, and wellbeing

Across multiple countries, GPs report intensifying workload and administrative burden, often without commensurate autonomy or team support. In England, newly trained resident doctors’ average contracted hours remain below full-time, a stable pattern over recent cohorts, whereas conversion rates from training to substantive roles have declined since 2021, signalling deteriorating job attractiveness at the point of entry.^30^ In Czechia, the modal exit age for women fell during 2020–22, consistent with early exit pressures linked to pandemic stress and regulatory change; meanwhile, many physicians continue beyond retirement but with reduced FTEs, eroding effective capacity even when headcount appears stable.^20^

Portugal’s decomposition further shows how policy decisions (eg, caps and the weighting of lists and the distribution of doctors across unit types) can inadvertently shift productivity downward, increasing the number of residents without an assigned GP despite ongoing recruitment rounds.^8^ Estonia has legislated that panels exceeding 2000 patients require the hiring of an additional GP, explicitly linking list size to quality and safety of care provision.^31^ Variation in consultation length might contribute to differences in job satisfaction among GPs. Reported averages range from 7 to 10 min in Spain to 9·8 min in the Netherlands, compared with 22·5 min in Sweden.^32^ Shorter consultation times might constrain comprehensive, patient-centred care, thereby increasing perceived pressure and reducing professional fulfilment.

### The pull of the private sector for GPs

In countries such as Italy, Portugal, and the UK, the private sector is now playing an increasingly prominent role in attracting primary care physicians away from traditional roles in public health systems, thereby reshaping workforce dynamics in general practice. Evidence from England illustrates how the expansion of privately funded general practice has created alternative employment opportunities for GPs, often characterised by different organisational models and patient mixes.^33^ At the same time, sustained workforce pressures in the public sector appear to reinforce these shifts, and evidence shows how recruitment and retention challenges in public general practice create incentives for doctors to seek alternative or supplementary employment outside the NHS.^29^

Comparative European research indicates that differences in remuneration, autonomy, workload, and organisational flexibility help to explain why primary care doctors might be attracted to private practice. In Sweden, systematic differences in service delivery models and incentives between public and private primary care providers appear to shape physician behaviour and employment preferences.^34^

More broadly, the phenomenon of dual practice—where doctors combine public and private work—has become a defining feature of many European health systems, even for primary care doctors.^35^ Country-specific analyses underline the ambivalence of dual practice as both a challenge and a coping mechanism for doctors and systems; evidence from Spain^36^ shows that dual practice can alleviate public-sector shortages but might also exacerbate inequalities and distort incentives. Similarly, an OECD study of Ireland^37^ highlights how private practice opportunities within and alongside public systems can attract doctors while complicating workforce planning and public-sector performance.

### A European pattern with national inflections

Although echoing what was observed in the USA and Commonwealth labour markets,^38^^,^^39^ the regional picture is not uniform: Nordic gatekeeping systems, such as those in Norway, Sweden and Denmark, with strong team-based models perform better on some indicators than fragmented arrangements that benefit specific interest groups; yet even high-performing settings report rural maldistribution and retirement bulges.^11^ The common threads are clear: weak primary care governance, low investment, insufficient data to manage FTE capacity in real time, and restricted progress on skill mix (eg, advanced nursing roles and pharmacist integration) that could relieve GPs of tasks not requiring medical training without compromising safety or quality of care.^21^

The contours of Europe’s GP workforce crisis are thus shaped by converging trends, attrition at entry, earlier exits, shrinking FTEs, and governance choices that constraint the throughput of primary care. Any credible strategy should simultaneously act on training, recruitment, retention, and work redesign, taking into account labour-market forces,^40^ and grounded in much better, FTE-sensitive data,^29^ if Europe is to rebuild the capacity of general practice for the next decade.

## Framing the policy challenge: a workforce lifecycle and labour-market approach

Our analysis suggests that Europe’s GP workforce crisis stems from cumulative weaknesses throughout the entire professional lifecycle rather than from any single failure. Insufficient undergraduate exposure restricts the pool of entrants;^27^ outdated numerus clausus policies cap the number of willing candidates;^41^ poorly designed early-career roles reduce conversion from training to practice;^14^ increasing workload and low autonomy fuel mid-career exits; and inadequate support for older clinicians accelerates retirement and reduces effective FTE capacity.^42^ These interacting pressures mean that stable GP headcounts can mask a decline in real capacity.

A labour-market perspective further clarifies these dynamics.^43^, ^44^, ^45^ Workforce supply is shaped by medical schools, trainees, professional associations, and practising doctors, whereas demand is determined by health systems, employers, insurers, and patients through remuneration, workload, contracts, and team organisation. Misalignment between supply and demand, such as expanding training without improving job quality, creates persistent shortages. These dynamics vary across Europe: some countries struggle primarily with recruitment, others with emigration, declining participation, or excessive administrative workload (table).

**Table:** GP workforce challenges, with country examples and supply-side and demand-side interventions

	Examples of selected countries	Supply-side interventions (medical schools, associations, and doctors)	Demand-side interventions (national health systems, primary care trusts, and private clinics)
An ageing GP population and failure to retain doctors in rural or challenging locations	France,^7^ Italy,^9^ and Spain^20^	Support local and rural recruitment and training of GPs and rural doctor quotas^10^^,^^46^	Increase funding for rural posting and infrastructure investment for rural practices^47^
GPs' migration to more lucrative labour markets was made easier by European integration	Czechia,^20^ Romania,^9^ Serbia,^48^ and Spain^7^	Tailor the national general practice curriculum specific to the training country’s health system;^48^ Continent-wide regulation protecting vulnerable markets.	Improve opportunities for career progression in sending countries^9^^,^^48^^,^^49^
Inability of the health system to fill vacancies in primary care	UK^8^^,^^12^	Allow fully trained new entrants (registrars) to practice and be employed in training surgeries^12^	Streamlining the deployment of primary care doctors^12^
Increased workload and risk of burnout for primary care practitioners	UK and Austria^9^^,^^19^	Enhancing workload management, increasing resources, fostering supportive work cultures, and implementing flexible working arrangements Use FTE GP instead of headcounts to allocate workloads sustainably^29^	Task shifting and task sharing; advanced roles for nurses in primary care to take on tasks and responsibilities and alleviate doctors’ workload^22^
Not enough medical students select general practice as their speciality of choice	All European countries	Longitudinal programmes in medical education, including clinical placements, peer-based learning community, GP mentorship programmes, GP placements, and compulsory primary care clerkships^10^^,^^28^	Introducing attractive primary practice models that reward career development opportunities, greater professional autonomy, peer support, and more flexibility in working patterns;^28^^,^^50^ Career pathways to promote and progress GPs into leadership and responsibility roles^51^
Outdated and inadequate numerus clausus policies penalise general practice	France^41^	Increasing numerus clausus ceilings for general practice only^41^	Financial and progression incentives for medical students to select primary care specialities^52^
Pay differentials between primary care and hospital specialities	Portugal, Czechia, and Romania	Medical associations to negotiate sliding remuneration scales harmonised across the continent or region^28^	Improving disproportionately working conditions and salary levels for general practice vis-à-vis hospital specialities
Physicians feeling overwhelmed with administrative work	All European countries^8^^,^^20^^,^^29^	Promoting medical education curricula focusing on data, management, and research^28^	New models of multiprofessional primary practice optimising skills^28^^,^^50^^,^^53^
The private sector is competing with public health-care systems to employ GPs	France, Portugal, and UK^29^^,^^54^	Statutory period of NHS primary care practice after graduation (New to Practice Fellowships) Regulation of private primary care services by a speciality council^29^^,^^54^	Measures to make NHS general practice more competitive than private sector alternatives, such as improving financial stability, increasing workforce flexibility, and adjusting contract structures; Returner and Refresher schemes are enhancing programmes to bring back GPs who have left the sector^55^^,^^56^
Urbanisation and geographical imbalances of the GP workforce^57^	France^58^	Supply-side incentives to medical schools to offer primary care residency experiences in basic medicine curricula^28^^,^^58^	Demand-side incentives to regional trusts to speed up the deployment of GPs and improve the conditions of practice in rural areas^28^

FTE=full-time equivalent. GP=general practitioner. NHS=National Health Service.

Effective solutions, therefore, cannot be uniform. Policies should reflect national governance arrangements, labour-market conditions, mobility patterns, remuneration structures, and rural distribution. At the same time, Europe requires a coherent, overarching strategy that links education, employment conditions, work organisation, and governance. The policy options that follow are framed within this lifecycle and labour-market approach (appendix p 6).

### Strengthening educational pathways into general practice

A resilient GP workforce begins with strong educational foundations. In many European countries, however, general practice remains insufficiently embedded in undergraduate medical training. Students often graduate with scarce exposure to primary care, shaping perceptions that favour hospital specialties. Making general practice a mandatory component of medical education, supported by longitudinal community placements, can improve understanding of the specialty and strengthen recruitment.^10^^,^^28^ Well-resourced academic departments of family medicine are essential to provide teaching, research capacity, and visible professional role models.

Postgraduate training is another crucial stage, but many countries struggle to fill GP residency posts or retain trainees. Training capacity should align with long-term workforce forecasts, whereas curricula should prepare doctors for the realities of modern primary care, including multimorbidity, continuity of care, community-oriented practice, and digital tools. Strong community placements and structured exposure to multidisciplinary teams improve preparedness and attractiveness. Rural shortages are especially acute, and evidence shows that training in rural settings increases the likelihood of long-term settlement; expanding rural pathways and mentorship programmes is therefore essential.^50^^,^^53^

Sustaining the workforce also requires viable academic and professional career structures. Opportunities in teaching, research, and clinical leadership strengthen the long-term attractiveness of general practice and support innovation within primary care systems. Ensuring GPs have equitable access to diagnostics and treatments is also crucial; when primary care clinicians face restrictions not applied to hospital specialists, professional status and job satisfaction decline.^9^^,^^48^^,^^49^

Strengthening educational pathways—from undergraduate exposure to postgraduate training and academic career development—enhances the prestige of general practice and increases conversion from training into long-term practice.

### Improving recruitment into general practice careers

Recruitment into general practice remains a major challenge across Europe. Although many countries train sufficient doctors, too few choose GP roles, and even fewer enter jobs that support long-term commitment. Evidence from Spain, Portugal, Czechia, and Romania shows that the problem stems less from a low interest rather than from policy-driven disincentives such as unstable contracts, low salaries, scarce support, and uneven post-distribution.^2^^,^^7^, ^8^, ^9^^,^^20^ Addressing these barriers requires targeted action at the point of entry, focusing on early-career roles, underserved areas, and the factors pushing doctors to emigrate.

Newly qualified GPs often face difficulties during the transition to practice. Heavy reliance on short-term contracts undermines job security and makes public-sector posts, already characterised by high workloads and inconsistent support, less attractive. Providing permanent, stable contracts is essential for improving recruitment.^12^^,^^30^ Competitive pay is equally important, as GP salaries lag behind those in hospital specialities, and intense workloads deter new graduates. Salary parity and clear progression pathways help to make GP roles financially viable.^28^^,^^29^^,^^54^

Strengthening early-career support is also crucial. Structured induction, supervision, and mentorship build confidence, consolidate skills, and reduce the risk of poorly supported doctors leaving the speciality or seeking opportunities abroad.^10^^,^^48^

Geographical maldistribution is a major contributor to Europe’s GP shortages, with rural, remote, and deprived areas consistently underserved even when national numbers are adequate. Addressing maldistribution requires targeted interventions that meet both professional and personal needs. Financial incentives, such as salary supplements, relocation grants, housing support, and childcare, can help to attract GPs, but they are insufficient without good working conditions. Modern facilities, safe out-of-hours environments, and reliable infrastructure are essential to making these posts viable.^46^^,^^47^

Exposure during training also plays a key role. Trainees who spend time in rural settings are more likely to return once qualified, so guaranteed rural rotations, structured placements, and mentorship programmes can reduce professional isolation and improve recruitment.^10^^,^^28^ When supported by strong governance and adequate staffing, these measures help in stabilising services in hard-to-staff regions.

Mobility further shapes workforce shortages. Many new GPs leave countries with poor working conditions, unstable contracts, and restricted opportunities for advancement. Improving job stability, workload, and governance is therefore central to reducing outward migration. Some GPs would consider returning if domestic conditions improved, so reintegration schemes, recognition of overseas experience, and opportunities for leadership or portfolio careers can support return migration, although care is needed to ensure this does not encourage populist, xenophobic narratives.^7^^,^^9^^,^^31^

Stronger European coordination is also needed to prevent workforce drain from countries with weak replacement pipelines. Shared planning, transparent mobility data, and EU-level dialogue can help in ensuring free movement does not undermine capacity in vulnerable systems.^11^^,^^59^

### Enhancing career progression, professional development, and retention

Strengthening the GP workforce requires not only attracting new entrants but also creating conditions that support doctors throughout their careers. Restricted career progression, reduced autonomy, and inflexible work patterns remain key drivers of dissatisfaction and early exit. Addressing these issues requires redesigned career structures, improved daily working conditions, and tailored support for mid-career and late-career clinicians whose continued participation is essential to maintaining capacity.^8^^,^^21^

Across Europe, GPs often do not have clear pathways for advancement, unlike many hospital specialists. Developing structured routes for senior clinical roles, leadership positions, partner-track opportunities, and accredited special interests can enhance job satisfaction and expand primary care’s capabilities. These pathways signal meaningful progression and help to retain experienced clinicians.

Working conditions are equally important. Restrictions on access to diagnostics, referrals, and treatments undermine GP autonomy and reduce job satisfaction. Ensuring equitable access across care settings supports evidence-based practice. Reducing administrative burdens, such as outdated sickness certification processes and inefficient documentation, can free up GPs' time for complex care and improve retention.^9^^,^^19^

Mid-career and late-career GPs are increasingly vulnerable to burnout and early retirement. Flexible options such as portfolio careers, combined clinical–academic posts, part-time work, and reduced on-call duties help in sustaining participation. Phased retirement models, with reduced clinical hours but continued involvement through mentoring or advisory roles, preserve expertise and maintain continuity of care.

Together, improved career progression, fair working conditions, and flexible retention strategies create a sustainable career environment. Redesigning the GP career journey, from early roles to senior leadership and gradual retirement, strengthens long-term workforce stability and helps ensure that general practice remains a viable and fulfilling profession.

### Redesigning work, expanding teams, and scaling up task sharing

Many of the pressures facing Europe’s GP workforce stem not only from clinician shortages but also from the organisation of work. In many systems, GPs handle most administrative tasks, routine clinical duties, and care coordination, creating unsustainable workloads even when headcounts appear stable. Experiences from Spain, Portugal, Czechia, and Romania show that restricted team structures, heavy bureaucracy, and rigid task boundaries contribute to burnout, early exits, and reduced full-time participation. Restoring capacity requires redesigning work to strengthen multidisciplinary teams, reduce administrative load, and align tasks more closely with skills.^10^^,^^22^

Multidisciplinary teams that integrate nurses, pharmacists, mental health practitioners, physiotherapists, social prescribers, and care coordinators can reduce GP workload and improve continuity of care. Examples from studies on Spanish GPs working abroad^7^ and on the dynamics of Portugal’s Family Health Units^8^ show how broader teams allow GPs to focus on higher-value clinical work. To avoid fragmentation, expanded teams need consistent training, robust governance, integrated records, and clearly defined responsibilities so that additional roles reduce rather than add to coordination demands.

Administrative overload is a major driver of attrition. Tasks such as prescription processing, documentation, triage, follow-up messaging, and certification often consume significant GP time, although they are suitable for trained administrative staff and, increasingly, automated digital platforms. Delegating these functions, improving e-prescription systems, and simplifying certification processes can significantly reduce bureaucracy, improve job satisfaction, and ease the early-career transition.

Effective task sharing requires matching responsibilities to each professional group's competencies. Preventive care, routine follow-up for stable chronic conditions, patient education, and some triage can be safely delegated when training and governance are strong. Protecting GP time for activities requiring advanced expertise, such as managing multimorbidity, acute undifferentiated illness, and longitudinal care, improves quality and reduces overload.^8^^,^^20^^,^^29^

Together, expanded teams, reduced bureaucracy, and clearer task alignment are central to rebuilding capacity. By redesigning work, health systems can ensure that GPs operate at the top of their licence, team members contribute effectively within their scope of practice, and patients receive more coordinated and sustainable care.

### Strengthening governance, data, and workforce planning

Effective governance and strong workforce planning are essential to addressing the structural drivers of Europe’s GP workforce crisis. Reforms to increase supply or improve retention only work if health systems can monitor workforce trends in real time, anticipate future needs, and align resources with population demand. Evidence from Portugal and Czechia shows the value of governance frameworks that use FTE staff data, adopt forward-looking planning tools, and implement regulatory mechanisms that balance workload, access, and equity.^8^^,^^20^

Traditional headcount-based indicators hide major variations in actual capacity, as reduced hours, early exits, and changing panel sizes often have a greater impact than clinician numbers alone. Real-time monitoring systems that track effective clinical hours, patient-to-GP ratios, and coverage allow earlier detection of pressures and more precise responses. Harmonised EU-level indicators on staffing (including full and part-time working), regional distribution, retirement risk, vacancies, and training performance would strengthen planning and facilitate cross-country comparisons.

Modern workforce planning should also move beyond static models. Forecasting tools need to account for ageing populations, multimorbidity, rising consultation rates, and declining FTE participation linked to burnout or part-time work. Dynamic modelling can guide adjustments to training capacity and distribution, and help to prevent chronic vacancies and enabling countries to respond proactively to waves of retirement and migration.^60^

Governance mechanisms should also ensure sustainable workloads. Funding models should reflect actual case-mix and incentivise comprehensiveness, continuity, and team-based care. Regulating panel sizes based on complexity and team composition can protect quality and prevent excessive workload compression. Contract structures should also incorporate equity: practices serving deprived or high-need populations require targeted incentives and stabilisation measures to prevent further maldistribution.

Together, improved governance, real-time workforce intelligence, and modern planning tools provide a foundation for lasting GP workforce reform. They ensure that interventions across training, recruitment, retention, and team-based care translate into durable improvements in primary care capacity.

### Leveraging demand-side reform to protect GP time and capacity

A sustainable GP workforce relies not only on training, recruitment, and career development but also on reshaping the conditions in which GPs work. Funding models, contracting arrangements, service delivery design, private-sector competition, and infrastructure all influence workload, job satisfaction, and effective clinical capacity. Strengthening these demand-side factors is essential to ensuring that GPs can provide safe, comprehensive care within systems that support rather than strain them.

Funding and contracting reforms are central. Many current models underestimate the intensity and complexity of primary care work. Aligning contracts with the core functions of general practice and rewarding continuity, preventive care, team-based models, and improved access allow practices to organise work more efficiently and reduces reliance on GP-only labour. Payment systems that support multidisciplinary teams and longer consultations for complex patients help in ensuring sustainability.^11^^,^^28^

Private-sector competition also shapes workforce stability. When public systems cannot match the autonomy, workload, or remuneration offered by private providers, they struggle to retain GPs. Strengthening public-sector conditions through greater stability, fair pay, stronger governance, and improved autonomy is the most effective way to prevent workforce drain. Measures such as minimum public service periods after publicly funded training might be used only in conjunction with genuine improvements in working conditions.^29^^,^^54^

Physical infrastructure remains a key enabling factor. Outdated facilities, inadequate consulting space, and poorly resourced out-of-hours services undermine recruitment and retention, especially in rural areas. Investment in safe, modern out-of-hours centres, team-ready primary care buildings, and digital tools, such as integrated electronic health records and automated administrative systems, reduces GP workload and supports effective task sharing.

Together, demand-side reforms to funding, contracting, private-sector regulation, and infrastructure are essential to protecting GP time and building a sustainable primary care system.^61^ By ensuring that employment conditions, payment models, and organisational environments enable rather than overwhelm clinicians, health systems strengthen the impact of supply-side interventions and support a resilient GP workforce.

### Policy packages tailored to the national context

Although Europe faces a shared GP workforce crisis, its causes and manifestations vary across health system contexts. Grouping countries into broad typologies helps illustrate these differences and guide policy responses.

In north-western European systems such as the UK and Germany, primary care institutions are relatively strong but face ageing GP cohorts, declining FTE participation, and rising workload pressures. Policy priorities include improving job design, reducing administrative burden, expanding multidisciplinary teams, and developing flexible late-career options.

Southern European systems, including those in Spain and Portugal, often produce sufficient numbers of doctors but struggle with outward migration, unstable employment conditions, and restricted career structures. Policies in these contexts should prioritise improved job stability, competitive remuneration, stronger academic departments, and expanded early-career support.

Eastern European systems face additional challenges, including rural shortages, underinvestment in infrastructure, and lower professional prestige for family medicine. Addressing these issues requires targeted rural recruitment strategies, modernised facilities, expanded rural training pathways, and strengthened governance.

Even high-performing primary care systems in Nordic countries and the Netherlands face rising demand, increasing multimorbidity, and growing workload complexity. These systems should expand multidisciplinary teams, improve data-driven workforce planning, and adapt funding models to reflect changing patient needs.

These examples show that Europe’s GP workforce crisis is not uniform. Effective reform requires tailored policy packages aligned with national institutional and labour-market realities while maintaining a shared commitment to strengthening primary care as the foundation of resilient health systems.

## A European roadmap for GP workforce renewal

First, we acknowledge that our analysis is constrained by limitations in the Joint Questionnaire dataset used to assess the GP workforce across Europe. In particular, inconsistencies between headcount and FTE measures limit comparability. Cross-country heterogeneity in definitions of GP or family doctor further reduces comparability, whereas variations in data completeness and reporting lag limit the ability to capture recent workforce changes.

Second, using a rapid review methodology to identify policy responses entails trade-offs compared with a full systematic review. Although appropriate for a fast-evolving policy context, the approach might have missed relevant studies, particularly in the grey literature, and increased the risk of selection bias due to streamlined screening and extraction processes. Third, reliance on a coordinated academic collection of country case studies introduces potential bias in both coverage and interpretation. Contributions might reflect the perspectives and expertise of participating researchers, with possible overrepresentation of countries with stronger academic networks.

Despite such limitations, our analysis strongly suggests that Europe’s GP workforce crisis cannot be solved through isolated reforms or simply by expanding training numbers. The crisis reflects systemic pressures across the professional lifecycle—from education and recruitment, to retention and late-career participation—and is shaped by labour-market conditions, governance, and the organisation of primary care work. Addressing such a crisis therefore requires a coordinated, market-informed, lifecycle-based strategy.

Reform should begin with stronger educational pathways, including sustained undergraduate exposure to general practice, expanded postgraduate training, and robust academic departments that enhance the speciality’s status. Recruitment policies should improve the conditions for newly qualified GPs through stable contracts, competitive remuneration, supervision, and clear career structures, whereas targeted incentives and improved infrastructure are needed to address persistent rural and regional shortages. Retention depends on better career progression, reduced administrative burden, and flexible mid-career and late-career roles that preserve expertise and maintain effective FTE capacity.

Sustainable reform also requires organisational and system-level change. Expanding multidisciplinary teams, redesigning workloads through task sharing, and strengthening workforce governance, supported by FTE-sensitive monitoring and modern workforce planning, can help to align capacity with population needs. Although policy responses should be tailored to diverse national contexts, a shared European vision is essential. By acting across education, employment, work organisation, and governance, countries can rebuild a resilient GP workforce and restore primary care as the foundation of accessible and equitable health systems. Responding to challenges in general practice, family medicine, and primary care is imperative for the future sustainability of the overall health system in each country.

## Declaration of interests

MM acted as President of the British Medical Association between 2022–23. SAB serves as the chair of the WONCA Europe Working Party on Policy Advocacy since 2023 and has received travel support from WONCA Europe. All other authors declare no competing interests.
