# Correction to *Lancet Prim Care* 2026; 2: 100166

**DOI:** 10.1016/j.lanprc.2026.100188

**Published:** 2026-06

*Russo G, McKee M, Zapata T, et al. The general practice workforce crisis in Europe: scale of the challenge and available policy responses.* Lancet Prim Care *2026;* ***2:*** *100166*—In this Health Policy paper, the list of professional organisations endorsing the policy document cited in reference 5 have been updated in the second paragraph of the Introduction. Additionally, the surname of co-author Alba Llop Girones has been corrected. These corrections have been made as of June 22, 2026.

