# Supplementary material for: The general practice workforce crisis in Europe: scale of the challenge and available policy responses
Source: Lancet Prim Care. 2026 Jun;2(6):None. doi: 10.1016/j.lanprc.2026.100166 (PMC13343396; doi:10.1016/j.lanprc.2026.100166)

THE LANCET
Primary Care

Supplementary appendix

This appendix formed part of the original submission and has been peer reviewed.
We post it as supplied by the authors.

Supplement to: Russo G, McKee M, Zapata T, et al. The general practice workforce crisis in Europe: scale of the challenge and available policy responses. *Lancet Prim Care* 2026.
https://doi.org/10.1016/j.lanprc.2026.100166

Supplementary material 1: Full list of papers selected it the rapid review

Full reference	Paper (short label)	Geographical focus	Study type
Abdullozoda, J., Yusufi, S., Nandi, S., Makhmudova, P., Bustamante, J.P., Langins, M., Llop-Gironés, A., Dastan, I., Olsavszky, V., Sultonov, S., Najmuddinova, Z., Azzopardi-Muscat, N., Zapata, T., 2024. Informing policy with health labour market analysis to improve availability of family doctors in Tajikistan. Hum Resour Health 22, 63. https://doi.org/10.1186/s12960-024-00946-5	Tajikistan GP labour market analysis	Tajikistan	Policy analysis
Armstrong, M.J., Wildman, J.M., Sowden, S., 2024. How to address the inverse care law and increase GP recruitment in areas of socioeconomic deprivation: a qualitative study of GP trainees' views and experiences in the UK. BJGP Open. https://doi.org/10.3399/BJGPO.2023.0201	UK GP trainees inverse care law	UK	Qualitative
Barnard, R., Spooner, S., Hubmann, M., Checkland, K., Campbell, J., Swinglehurst, D., 2024. The hidden work of general practitioners: An ethnography. Social Science & Medicine 350, 116922. https://doi.org/10.1016/j.socscimed.2024.116922	GP hidden work ethnography	UK	Qualitative
Barry, T., Batt, A., Agarwal, G., Booker, M., Casey, M., McCombe, G., 2022. Potential for Paramedic roles in Irish General Practice: A qualitative study of stakeholder's perspectives. HRB Open Res 5, 40. https://doi.org/10.12688/hrbopenres.13545.2	Paramedics in Irish general practice	Ireland	Qualitative
Bes, J.M., Flinterman, L.E., González, A.I., Batenburg, R.S., 2023. Recruitment and retention of general practitioners in European medical deserts: a systematic review. Rural and Remote Health 23. https://doi.org/10.22605/RRH7477	GP retention rural deserts review	European region	Review
Bi, Y.-N., Liu, Y.-A., 2023. GPs in UK: From Health Gatekeepers in Primary Care to Health Agents in Primary Health Care. Risk Manag Healthc Policy 16, 1929–1939. https://doi.org/10.2147/RMHP.S416934	UK GP role transformation	UK	Conceptual
Burgmann, S., Huter, S., Mayerl, H., Paier-Abuzahra, M., Siebenhofer, A., 2023. Facilitators and barriers in general practitioners' choice to work in primary care units in Austria: a qualitative	GP recruitment Austria	Austria	Qualitative
Calderón-Larrañaga, S., González-De-La-Fuente, Á., Espinosa-González, A.B., Casado-Vicente, V., Brito-Fernandes, Ó., Klazinga, N., Kringos, D., 2024. What can we learn from general practitioners who left Spain? A mixed methods international study. Hum Resour Health 22, 9. https://doi.org/10.1186/s12960-023-00888-4	GPs leaving Spain	Spain	Mixed-methods
Chilvers, R., Richards, S.H., Fletcher, E., Aylward, A., Dean, S., Salisbury, C., Campbell, J., 2019. Identifying policies and strategies for general practitioner retention in direct patient care in the United Kingdom: a RAND/UCLA appropriateness method panel study. BMC Family Practice 20, 130. https://doi.org/10.1186/s12875-019-1020-x	UK GP retention policies RAND/UCLA	UK	Quantitative
Ciotlăuș, S., Ungureanu, M.I., Oprescu, F., 2025. Adverse working conditions in Romanian out-of-hours primary care (OOH-PC): an interview study. Hum Resour Health 23, 37. https://doi.org/10.1186/s12960-025-00985-6	Romanian OOH primary care	Romania	Qualitative
Costa, E., Pestana, J., Barros, P.P., 2024. Primary health care coverage in Portugal: the promise of a general practitioner for all. Hum Resour Health 22, 55. https://doi.org/10.1186/s12960-024-00936-7	Portugal GP coverage	Portugal	Quantitative
Danhieux, K., Buffel, V., Pairon, A., Benkheil, A., Remmen, R., Wouters, E., van Olmen, J., 2020. The impact of COVID-19 on chronic care according to providers: a qualitative study among primary care practices in Belgium. BMC Fam Pract 21, 255. https://doi.org/10.1186/s12875-020-01326-3	COVID chronic care Belgium	Belgium	Qualitative
de Oliveira, A.P.C., Dussault, G., 2024. Interventions to attract medical students to a career in primary health care services in the European Union and peripheral countries: a scoping review. Hum Resour Health 22, 69. https://doi.org/10.1186/s12960-024-00943-8	EU medical student attraction scoping	European region	Review

Full reference	Paper (short label)	Geographical focus	Study type
Drennan, V.M., Halter, M., Wheeler, C., Nice, L., Brearley, S., Ennis, J., Gabe, J., Gage, H., Levenson, R., de Lusignan, S., Begg, P., Parle, J., 2019. What is the contribution of physician associates in hospital care in England? A mixed methods, multiple case study. BMJ Open 9, e027012. https://doi.org/10.1136/bmjopen-2018-027012	Physician associates England hospital	UK	Mixed-methods
Fernemark, H., Karlsson, N., Skagerström, J., Seing, I., Karlsson, E., Brulin, E., Nilsen, P., 2024. Psychosocial work environment in Swedish primary healthcare: a cross-sectional survey of physicians' job satisfaction, turnover intention, social support, leadership climate and change fatigue. Hum Resour Health 22, 70. https://doi.org/10.1186/s12960-024-00955-4	Swedish primary care work environment	Sweden	Survey
Forbes, L.J., Forbes, H., Sutton, M., Checkland, K., Peckham, S., 2020. Changes in patient experience associated with growth and collaboration in general practice: observational study using data from the UK GP Patient Survey. Br J Gen Pract 70, e906–e915. https://doi.org/10.3399/bjgp20X713429	UK GP patient experience	UK	Quantitative
García-Prado, A., González, P., 2024. [Dual practice in the Spanish health system: problem or solution? SESPAS Report 2024]. Gac Sanit 38 Suppl 1, 102379. https://doi.org/10.1016/j.gaceta.2024.102379	Dual practice Spain report	Spain	Policy analysis
Gefaell Larrondo, I., Ares-Blanco, S., Guisado-Clavero, M., Mira, J.J., Pérez Esteve, C., Adler, L., Bensemmane, S., Kostić, M., Mortsiefer, A., Petrazzuoli, F., Gómez-Bravo, R., Astier-Peña, P., 2026. Strengthening Primary Health Care in Europe: A Delphi study towards accessibility, equity and continuity of care. Eur J Gen Pract 32, 2619226. https://doi.org/10.1080/13814788.2026.2619226	European primary care Delphi study	European region	Qualitative
Glenngård, A.H., 2023. Exploring differences between public and private providers in primary care: findings from a large Swedish region. Health Econ Policy Law 18, 219–233. https://doi.org/10.1017/S1744133122000251	Sweden public vs private care	Sweden	Quantitative
Groenewegen, P.P., Bosmans, M.W.G., Boerma, W.G.W., Spreeuwenberg, P., 2020a. The primary care workforce in Europe: a cross-sectional international comparison of rural and urban areas and changes between 1993 and 2011. Eur J Public Health 30, iv12–iv17. https://doi.org/10.1093/eurpub/ckaa125	European GP workforce comparison	European region	Case-study
Gunja, M., Gumas, E., Williams II, R., Doty, M., Shah, A., Fields, K., 2022. Stressed Out and Burned Out: The Global Primary Care Crisis. The Commonwealth Fund, Washington (DC). https://doi.org/10.26099/j2ag-mx88	Global primary care crisis	Worldwide, including Europe	Review
Havelková, T., Šídlo, L., 2025. Working life expectancy of physicians: the case of primary care physicians in Czechia. Hum Resour Health 23, 9. https://doi.org/10.1186/s12960-025-00978-5	Czech physician work expectancy	Czechia	Quantitative
Hodes, S., Hussain, S., Panja, A., Welch, E., Shire, R., 2022. When part time means full time: the GP paradox. BMJ 377, o1271. https://doi.org/10.1136/bmj.o1271	GP paradox part-time work UK	UK	Conceptual
Hutchinson, J., Gibson, J., Kontopantelis, E., Checkland, K., Spooner, S., Parisi, R., Sutton, M., 2024. Trends in full-time working in general practice: a repeated cross-sectional study. Br J Gen Pract 74, e652–e658. https://doi.org/10.3399/BJGP.2023.0432	UK GP full-time trends	UK	Quantitative
Jager, A., Harris, M., Terry, R., 2023. The challenges faced by early career international medical graduates in general practice and opportunities for supporting them: a rapid review. BJGP Open 7. https://doi.org/10.3399/BJGPO.2023.0012	IMGs GP careers rapid review	European region	Review
Jatić, Z., Smlatic, E., McGowan, M., Erkočević, H., Hasanović, E., Trifunović, N., 2023. Family Physicians' Perceptions of Primary Health Care Use in Bosnia and Herzegovina during the Covid-19 Pandemic, a Cross-sectional Study. Acta Med Acad 52, 13–23. https://doi.org/10.5644/ama2006-124.397	Bosnia primary care COVID	Bosnia & Herzegovina	Survey

Full reference	Paper (short label)	Geographical focus	Study type
Jobalayeva, B., Khismetova, Z., Glushkova, N., Kozhekenova, Z., Abzaliyeva, A., Berikuly, D., Semenova, Y., 2024. The impact of incentive scheme on rural healthcare workforce availability: a case study of Kazakhstan. Hum Resour Health 22, 23. https://doi.org/10.1186/s12960-024-00905-0	Kazakhstan rural incentives	Kazakhstan	Case-study
Joyce, P., Alexander, L., 2023. A survey exploring factors affecting employment of physician associates in Ireland. Ir J Med Sci 192, 2041–2046. https://doi.org/10.1007/s11845-022-03255-9	Ireland physician associates survey	Ireland	Survey
Kuhlmann, E., Falkenbach, M., Brînzac, M.G., Correia, T., Panagioti, M., Rechel, B., Sagan, A., Santric-Milicevic, M., Ungureanu, M.-I., Wallenburg, I., Burau, V., 2024. Tackling the primary healthcare workforce crisis: time to talk about health systems and governance—a comparative assessment of nine countries in the WHO European region. Hum Resour Health 22, 83. https://doi.org/10.1186/s12960-024-00965-2	WHO Europe workforce crisis	European region	Policy analysis
Marshall, M., Ikpoh, M., 2022. The workforce crisis in general practice. Br J Gen Pract 72, 204–205. https://doi.org/10.3399/bjgp22X719213	UK GP workforce crisis	UK	Conceptual
McElhinney, Z., Kennedy, C., 2021. By accident or design? An exploration of the career pathways, experiences and identities of academic GPs using composite narratives. Education for Primary Care 32, 266–271. https://doi.org/10.1080/14739879.2021.1894991	Academic GP narratives UK	UK	Qualitative
McKee, M., Vaughan, L.K., Russo, G., 2025. A contentious intervention to support the medical workforce: a case study of the policy of introducing physician associates in the United Kingdom. Hum Resour Health 23, 4. https://doi.org/10.1186/s12960-024-00966-1	Physician associates UK policy	UK	Policy analysis
Meyer, J., Gjessing, S., Kristensen, J.K., Flessa, S., 2023. Attitudes of medical students and junior physicians towards working self-employed in private practice in Northern and Western Europe: a systematic review. J Public Health (Berl.) 31, 1759–1772. https://doi.org/10.1007/s10389-022-01760-w	Europe private practice attitudes review	European region	Review
Mueller, M., Socha-Dietrich, K., 2020. Reassessing private practice in public hospitals in Ireland: An overview of OECD experiences. OECD Health Working Papers. https://doi.org/10.1787/111171d3-en	Ireland private practice OECD	Ireland	Policy analysis
Mughal, F., Mallen, C.D., McKee, M., 2021. The impact of COVID-19 on primary care in Europe. The Lancet Regional Health – Europe 6. https://doi.org/10.1016/j.lanepe.2021.100152	COVID impact on primary care Europe	European region	Review
Muscat, N.A., Lazëri, L., Zapata, T., Kluge, H., 2025. Protecting the mental health of the health and care workforce in Europe: a strategic investment. Lancet Reg Health Eur 57, 101489. https://doi.org/10.1016/j.lanepe.2025.101489	European workforce mental health	European region	Review
Nussbaum, C., Massou, E., Fisher, R., Morciano, M., Harmer, R., Ford, J., 2021. Inequalities in the distribution of the general practice workforce in England: a practice-level longitudinal analysis. BJGP Open 5. https://doi.org/10.3399/BJGPO.2021.0066	England GP distribution inequalities	UK	Quantitative
Oleszczyk, M., Stepanovič, A., Král, N., Seifert, B., Švab, I., Krzysztoń, J., Jagiełła, N., Windak, A., n.d. How far on the road? The role of family medicine/general practice in 10 Central and Eastern European countries: A mixed-method study. Eur J Gen Pract 31, 2594292. https://doi.org/10.1080/13814788.2025.2594292	CEE family medicine comparison	European region	Mixed-methods
Owen, K., Hopkins, T., Shortland, T., Dale, J., 2019. GP retention in the UK: a worsening crisis. Findings from a cross-sectional survey. BMJ Open 9, e026048. https://doi.org/10.1136/bmjopen-2018-026048	UK GP retention survey	UK	Survey
Paier-Abuzahra, M., Posch, N., Jeitler, K., Semlitsch, T., Radl-Karimi, C., Spary-Kainz, U., Horvath, K., Siebenhofer, A., 2024a. Effects of task-shifting from primary care physicians to nurses: an overview of systematic reviews. Hum Resour Health 22, 74. https://doi.org/10.1186/s12960-024-00956-3	Task shifting systematic reviews	European region	Review

Full reference	Paper (short label)	Geographical focus	Study type
Paier-Abuzahra, M., Posch, N., Spary-Kainz, U., Radl-Karimi, C., Semlitsch, T., Jeitler, K., Siebenhofer, A., 2024b. Effects of task shifting from primary care physicians to nurses: a protocol for an overview of systematic reviews. BMJ Open 14, e078414. https://doi.org/10.1136/bmjopen-2023-078414	Task shifting protocol	European region	Review
Palmer, W.L., Rolewicz, L., Tzortziou Brown, V., Russo, G., 2025. A hole in the bucket? Exploring England's retention rates of recently qualified GPs. Human Resources for Health 23, 14. https://doi.org/10.1186/s12960-025-00980-x	England GP retention trends	UK	Quantitative
Pestana, J., Frutuoso, J., Costa, E., Fonseca, F., 2024. Heterogeneity in physician's job preferences in a dual practice context – Evidence from a DCE. Social Science & Medicine 343, 116551. https://doi.org/10.1016/j.socscimed.2023.116551	Portugal GP preferences DCE	Portugal	Survey
Petrazzuoli, F., Collins, C., Van Poel, E., Tatsioni, A., Streit, S., Bojaj, G., Asenova, R., Hoffmann, K., Gabrani, J., Klemenc-Ketis, Z., Rochfort, A., Adler, L., Windak, A., Nessler, K., Willems, S., 2023. Differences between Rural and Urban Practices in the Response to the COVID-19 Pandemic: Outcomes from the PRICOV-19 Study in 38 Countries. International Journal of Environmental Research and Public Health 20, 3674. https://doi.org/10.3390/ijerph20043674	PRICOV-19 38 countries	European region	Quantitative
Pettigrew, L.M., Bharmal, A.V., Akl, S., Exley, J., Allen, L.N., Petersen, I., Cromwell, D.A., Mays, N., 2025. Trends in the shortfall of English NHS general practice doctors: repeat cross sectional study. BMJ 390, e083978. https://doi.org/10.1136/bmj-2024-083978	England GP shortage trends	UK	Quantitative
Pols, D.H.J., Kamps, A., Runhaar, J., Elshout, G., van Halewijn, K.F., Bindels, P.J.E., Stegers–Jager, K.M., 2023. Medical students' perception of general practice: a cross-sectional survey. BMC Med Educ 23, 103. https://doi.org/10.1186/s12909-023-04064-z	Medical student perceptions	European region	Survey
Razai, M.S., Majeed, A., 2022. General Practice in England: The Current Crisis, Opportunities, and Challenges. The Journal of Ambulatory Care Management 45, 135. https://doi.org/10.1097/JAC.0000000000000410	GP crisis England	UK	Conceptual
Russell, D., Mathew, S., Fitts, M., Liddle, Z., Murakami-Gold, L., Campbell, N., Ramjan, M., Zhao, Y., Hines, S., Humphreys, J.S., Wakerman, J., 2021. Interventions for health workforce retention in rural and remote areas: a systematic review. Hum Resour Health 19, 103. https://doi.org/10.1186/s12960-021-00643-7	Rural workforce retention review	Worldwide, including Europe	Review
Russo, G., Perelman, J., Zapata, T., Šantrić-Milićević, M., 2023. The layered crisis of the primary care medical workforce in the European region: what evidence do we need to identify causes and solutions? Human Resources for Health 21, 55. https://doi.org/10.1186/s12960-023-00842-4	European primary care workforce crisis	European region	Conceptual
Sinnott, C., Dorban-Hall, B., Dixon-Woods, M., 2023. Tackling the crisis in general practice. BMJ 381, p966. https://doi.org/10.1136/bmj.p966	UK GP crisis	UK	Conceptual
Siriwardena, A.N., Botan, V., Williams, N., Emerson, K., Kameen, F., Pope, L., Freeman, A., Law, G., 2023. Performance of ethnic minority versus White doctors in the MRCGP assessment 2016–2021: a cross-sectional study. Br J Gen Pract 73, e284–e293. https://doi.org/10.3399/BJGP.2022.0474	MRCGP ethnic minority performance	UK	Survey
Stark, S., Schaubroeck, E., Kluge, M., Burggraf, L., Roos, M., Borowski, E., Van Poel, E., Willems, S., Kühlein, T., Hueber, S., Werner, F., 2023. "The measures taken by the government overburdened the daily practice" - insights of the PRICOV-19 study on German general practitioners in times of COVID-19. BMC Prim Care 24, 207. https://doi.org/10.1186/s12875-023-02115-4	German GP COVID experience	Germany	Qualitative
Stobbe, E.J., Groenewegen, P.P., Schäfer, W., 2021. Job satisfaction of general practitioners: a cross-sectional survey in 34 countries. Hum Resour Health 19, 57. https://doi.org/10.1186/s12960-021-00604-0	GP job satisfaction 34 countries	European region	Survey

Full reference	Paper (short label)	Geographical focus	Study type
Tu, K., Kristiansson, R.S., Gronsbell, J., Lusignan, S. de, Flottorp, S., Goh, L.H., Hallinan, C.M., Hoang, U., Kang, S.Y., Kim, Y.S., Li, Z., Ling, Z.J., Manski-Nankervis, J.-A., Ng, A.P.P., Pace, W.D., Wensaas, K.-A., Wong, W.C., Stephenson, E., 2022. Changes in primary care visits arising from the COVID-19 pandemic: an international comparative study by the International Consortium of Primary Care Big Data Researchers (INTRePID). BMJ Open 12, e059130. https://doi.org/10.1136/bmjopen-2021-059130	International primary care big data	Worldwide, including Europe	Quantitative
Vaes, B., Vos, B., Foidart, M., De Schreye, R., Schrans, D., Philips, H., Aertgeerts, B., Doggen, K., 2022. Burden of COVID-19 on primary care in Belgium: a prospective nationwide observational study from March to August 2020. Arch Public Health 80, 250. https://doi.org/10.1186/s13690-022-01003-0	Belgium COVID primary care burden	Belgium	Quantitative
Velgan, M., Vanderheyde, T., Kalda, R., Michels, N., 2023. Driving forces of GPs' migration in Europe: an exploratory qualitative study. BJGP Open 7. https://doi.org/10.3399/BJGPO.2022.0132	GP migration Europe	European region	Qualitative
Wanat, M., Hoste, M., Gobat, N., Anastasaki, M., Böhmer, F., Chlabicz, S., Colliers, A., Farrell, K., Karkana, M.-N., Kinsman, J., Lionis, C., Marcinowicz, L., Reinhardt, K., Skoglund, I., Sundvall, P.-D., Vellinga, A., Verheij, T.J., Goossens, H., Butler, C.C., Velden, A. van der, Anthierens, S., Tonkin-Crine, S., 2021. Transformation of primary care during the COVID-19 pandemic: experiences of healthcare professionals in eight European countries. Br J Gen Pract 71, e634–e642. https://doi.org/10.3399/BJGP.2020.1112	COVID transformation 8 countries	European region	Qualitative
Weinmayr, L.-M., Zwierlein, R., Steinhäuser, J., 2020. Modifiable determinants for the success or failure of inter-physician collaboration in group practices in Germany - a qualitative study. BMC Fam Pract 21, 276. https://doi.org/10.1186/s12875-020-01349-w	GP collaboration Germany	Germany	Qualitative
Williams, G.A., Jacob, G., Rakovac, I., Scotter, C., Wismar, M., 2020. Health professional mobility in the WHO European Region and the WHO Global Code of Practice: data from the joint OECD/EUROSTAT/WHO-Europe questionnaire. Eur J Public Health 30, iv5–iv11. https://doi.org/10.1093/eurpub/ckaa124	European health professional mobility	European region	Quantitative
Zakarija-Grković, I., Vrdoljak, D., Cerovečki, V., 2018. What can we Learn from each other about Undergraduate Medical Education in General Practice/Family Medicine? Zdr Varst 57, 148–154. https://doi.org/10.2478/sjph-2018-0019	GP education undergraduate study	Slovenia	Survey
Żuk, Piotr, Żuk, Paweł, Lisiewicz-Jakubaszko, J., 2019. Labour migration of doctors and nurses and the impact on the quality of health care in Eastern European countries: The case of Poland. The Economic and Labour Relations Review 30, 307–320. https://doi.org/10.1177/1035304619847335	Poland doctor migration impact	Poland	Case-study

Supplementary material 2: Visual representation of bottlenecks and policy options for strengthening the primary care medical workforce in Europe

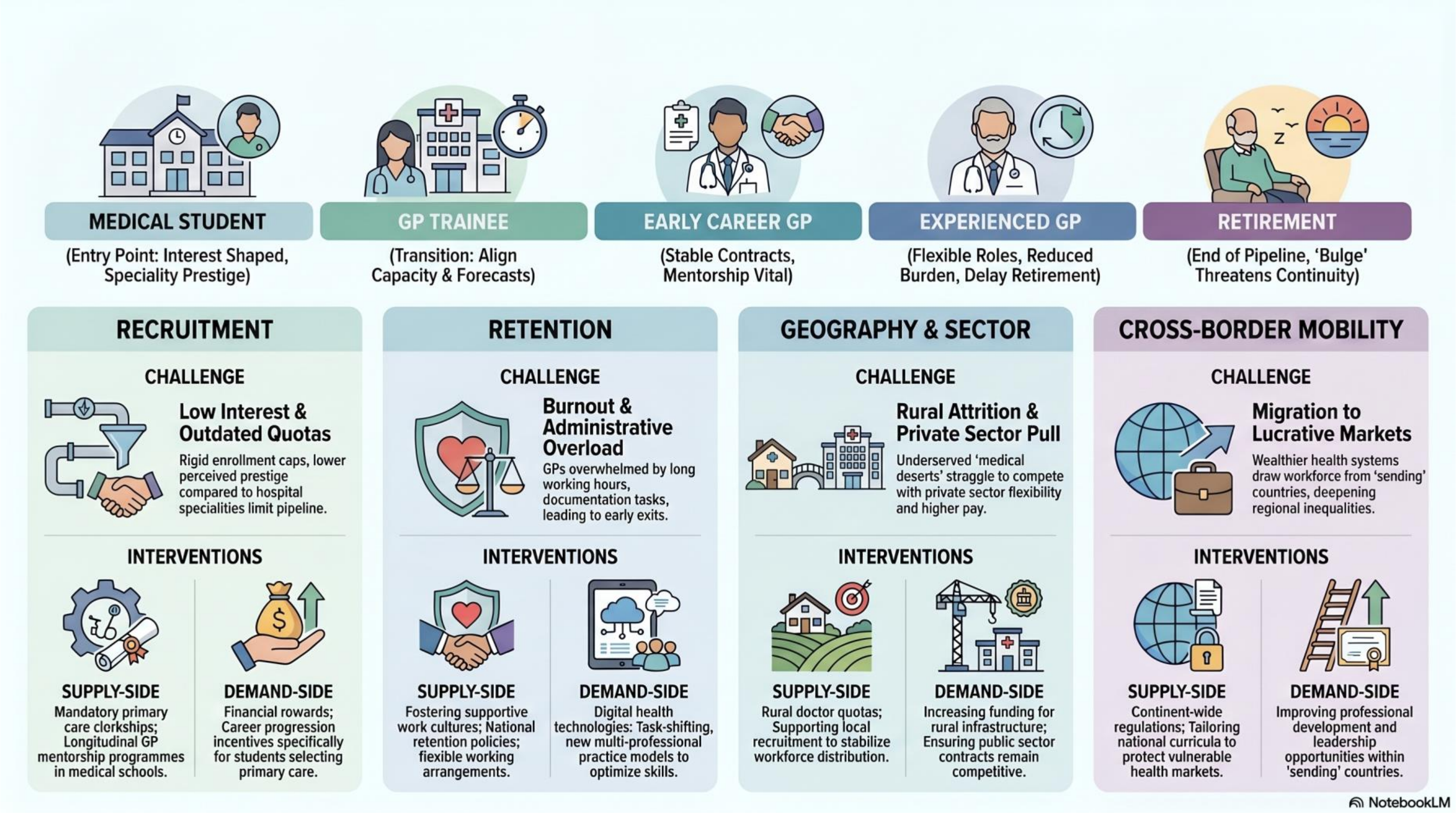

Supplement: Supplementary appendix [file mmc1.pdf]
